# Complement C7 (C7), a Potential Tumor Suppressor, Is an Immune-Related Prognostic Biomarker in Prostate Cancer (PC)

**DOI:** 10.3389/fonc.2020.01532

**Published:** 2020-08-25

**Authors:** Zhao Chen, Xin Yan, Guo-Wei Du, Kurerban Tuoheti, Xiao-Jie Bai, Hua-Hui Wu, Ren-Jie Zhang, Guan-Fa Xiao, Tong-Zu Liu

**Affiliations:** Department of Urology, Zhongnan Hospital of Wuhan University, Wuhan, China

**Keywords:** prostate cancer, prognostic biomarker, tumor immune microenvironment, copy number variations, molecule drugs

## Abstract

**Objectives:** Prostate cancer (PC) is the second most frequent tumor in men, which has a high recurrence rate and poor prognosis. Therefore, this study aimed to identify novel prognostic biomarkers and therapeutic targets for immunotherapy and small molecule drugs for PC treatment.

**Materials and Methods:** The Estimation of Stromal and Immune cells in Malignant Tumor tissues using Expression data (ESTIMATE) algorithm was applied to calculate immune scores and stromal scores of TCGA-PRAD data. Differentially expressed genes (DEGs) were identified using R package “limma.” GO, KEGG, and DO analyses were performed to analyze DEGs. Overall survival and disease-free survival analyses were conducted for hub gene identification. To validate the hub gene at the mRNA and protein expression levels, genetic alterations were measured, and CCLE and Cox regression analyses were performed. Connectivity map (CMap) analysis and GSEA were performed for drug exploration and function analysis, respectively.

**Results:** Immune scores ranged from −1795.98 to 2339.39, and stomal scores ranged from −1877.60 to 1659.96. In total, 45 tumor microenvironment (TME)-related DEGs were identified, of which Complement C7 (C7) was selected and validated as a hub gene. CMap analysis identified six small molecule drugs as potential agents for PC treatment. Further analysis demonstrated that C7 expression was significantly correlated with clinical T, pathological N, and immune infiltration level.

**Conclusions:** In conclusion, of the 45 TME-related DEGs, C7 was shown to correlate with PC prognosis in patients, indicating it as a novel prognostic biomarker and immunotherapy target in PC. Additionally, six small molecule drugs showed strong therapeutic potential for PC treatment.

## Introduction

Prostate cancer (PC) is the second most frequent tumor in men, with a high recurrence rate and age dependence (1). In 2018, 1,276,106 new cases of PC and 358,989 associated deaths occurred worldwide (1). Although serum PSA, CT, and magnetic resonance examination have been widely used for PC diagnosis, these methods do not show high accuracy and specificity (2). Thus, novel biomarkers to accurately diagnose PC are needed.

Recently, more studies have shown that immunotherapy could treat cancers effectively and safely (3–5). With the development of immunotherapy for cancers, more and more researchers focus on finding out more accurate therapeutic targets for immune treatment (6, 7). Thus, in this study, we aimed to screen some novel diagnostic and prognostic biomarkers for PC and therapeutic targets for immunotherapy.

The Cancer Genome Atlas (TCGA), a landmark cancer genomics program, molecularly characterized over 20,000 primary cancer and matched normal samples spanning 33 cancer types. Thus, we retrieved PC samples from TCGA-PRAD (prostate adenocarcinoma) data, which had more data, abundant information and contents characteristics. To the best of our knowledge, this study is the first to apply the Estimation of Stromal and Immune cells in Malignant Tumor tissues using Expression data (ESTIMATE) algorithm for immune score and stromal score calculation of each PC case from TCGA-PRAD data (8). Then, based on immune scores and stromal scores for PC samples, we attempted to identify and validate some novel immune-related prognostic biomarkers in PC.

In conclusion, our finding indicated that C7 might be a novel prognostic biomarker and therapeutic target for immunotherapy in PC. In addition, six molecule drugs were identified, which showed strong therapeutic potential for PC treatment.

## Materials and Methods

### PC Studies Collection and Data Preprocessing

First, gene expression profiles of mRNA of PC samples (TCGA-PRAD data) were retrieved from The Cancer Genome Atlas (TCGA) database (https://genomecancer.ucsc.edu/). Briefly, 495 PC cases with complete clinical information were included in this study. Using the Gene Expression Omnibus (GEO) database (http://www.ncbi.nlm.nih.gov/geo/), GSE116918 (of GPL25318) (9), GSE16560 (10) of GPL5474, and GSE70770 (11, 12) of GPL6801 were downloaded for validation. In total, 248 PCs from GSE116918 with complete survival information, 281 PCs from GSE16560 with survival information, and 204 PCs from GSE70770 with complete clinical information were included in this study.

**Figure 1:**
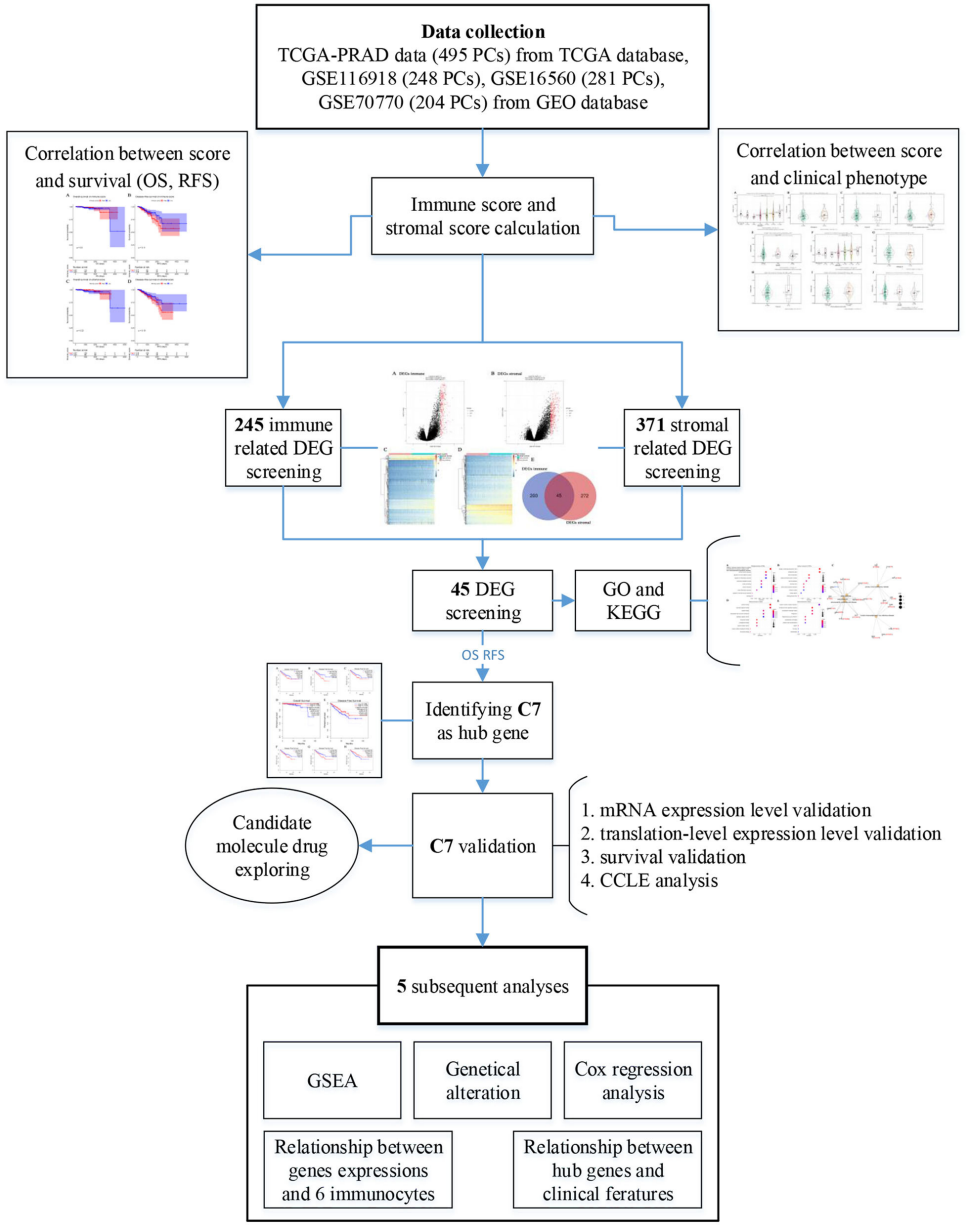
Flow diagram of data preparation, processing, analysis, and validation in this study.

Figure 1 provided a step-by-step flowchart of this study. In brief, all data were preprocessed before analysis. For the TCGA-PRAD data displayed as count number, normalized, and log2 transformation were performed using R package “DEseq.2” (13). Moreover, normalization and transformation of GSE116918 and GSE70770 were conducted using package “affy” (14) in R software, which were shown as raw expression data.

### Calculation of Immune and Stromal Scores and Correlation With Clinical Phenotype

Using the ESTIMATE algorithm, immune, and stromal scores of each case from the TCGA database were calculated by R package “estimate” (8). To explore the correlation between immune score or stromal score and clinical phenotype, the immune score levels (or stromal score levels) by gender (female vs. male), neoplasm histological grade (Gx, G1, G2, G3, G4), laterality (left vs. right), pathological stage (I, II, III, IV), and neoplasm cancer status (tumor-free vs. tumor) were assessed using boxplots. Subsequently, 495 PCs were divided into high- and low-immune score groups based on the median immune score. Similarly, cases were divided into high- and low-stromal score groups. Overall survival (OS) and disease-free survival (RFS) analyses were conducted to explore the relationship between immune score (or stromal score) and survival using R package “survival” (15). Unpaired *t*-test [or One-way Analysis of Variance (ANOVA) test] was performed to measure statistical significance.

### Differentially Expressed Gene (DEG) Identification and Hub Gene Identification

Immune- and stromal-related genes were screened to identify DEGs in this study. To detect immune-related DEGs, we first divided 495 PC cases into high- and low-immune groups based on the median immune score, followed by DEG screening using “limma” (16) package in R. Similarly, for stromal-related DEGs, 495 samples were divided into two groups (high- and low-stromal groups), and DEGs were screened by “limma.” Genes reaching the standard (Adjust *P* < 0.05 and | log2 fold change (FC) | ≥ 1.0) were considered as DEGs. Further, overlapping immune- and stromal-related DEGs were selected for subsequent analysis. Gene Ontology (GO) (17) enrichment analysis, Kyoto encyclopedia of Genes and Genomes (KEGG) (18) pathway analysis, and Disease Ontology (DO) (19) were performed through “clusterProfiler” (20) in R software to identify potential functions of DEGs and highly correlated diseases. Gene sets were considered significant when *P* < 0.05. In order to screen out hub genes among the DEGs, two survival analyses, including OS and RFS, were performed for each DEG by Gene Expression Profiling Interactive Analysis (GEPIA) (http://gepia.cancer-pku.cn/) (21). Only DEGs showing significant *P-*values in both survival analyses were considered to be correlated with survival and prognosis of patients with PC, which were regarded as hub genes in this study.

### Exploration of Hub Gene Expression in Normal Tissues and Tumors at Different Stages

First, we compared expression levels of hub genes in tumors and normal tissues by GEPIA. Based on GSE116918 and GSE70770 with complete T stage information, T stage (T1, T2, T3, and T4) boxplots were generated (GSE116918). Additionally, clinical T stage (T1, T2, and T3) and pathology T stage (T1, T2, and T3) boxplots were plotted. Unpaired *t-*test and One-way Analysis of Variance (ANOVA) test were conducted to measure statistical significance.

### Hub Gene Protein Expression and Survival Validation and Genetic Alteration

Following selection of hub genes, their mRNA expression levels in PC and normal tissues were compared using the Oncomine database (https://www.oncomine.org/) (22). mRNA expression and copy number variation (CNV) levels of hub genes in 40 tumor types were explored using the CCLE database (https://portals.broadinstitute.org/ccle/). Based on GSE116918, biochemical failure-free survival (BFFS) and metastasis free survival (MFS) analyses for hub genes were performed to validate the prognostic value. Similarly, based on GSE16560, OS of patients with PC was assessed. Further, protein expression levels of hub genes were validated using The Human Protein Atlas database (https://www.proteinatlas.org/) (23). Student *t-*test was used to measure statistical significance. By using the CBio Cancer Genomics Portal (http://www.cbioportal.org/) (24, 25), genetic alteration of hub genes was examined. Combined with relative mRNA expression levels, the correlation with copy number variations (CNVs) of hub genes was explored. Following, cases were divided into with and without alterations groups, and survival analyses (OS and RFS) were conducted to explore the association between mutations and survival of PC patients.

### Associations Between Hub Genes and Clinical Features of PC Patients

Based on TCGA-PRAD data, 495 PCs were divided into high- (*n* = 248) and low- (*n* = 247) expression groups by evaluating the median value of hub gene expression. Subsequently, χ2-test or ANOVA was performed to measure the relationships between hub gene expressions and clinical features (age, laterality, clinical M, clinical T, pathological N, pathological T, and neoplasm cancer status). Expression levels of hub genes and clinical features (age, laterality, Gleason score, clinical M, clinical T, pathological N, pathological T) were also included for univariable Cox analysis of overall survival (OS) using TCGA-PRAD data. Features with *P* < 0.05 were included for multivariate Cox analysis.

### Correlation Between Hub Genes and Immune Microenvironment

Using TIMER (https://cistrome.shinyapps.io/timer/) (26), the correlation between hub gene expression and the abundance of immune infiltrates was explored. Six types of tumor-infiltrating immune cells (B cells, CD8+ T cells, CD4+ T cells, macrophages, neutrophils, and dendritic cells) were included in this analysis. Hub genes with |correlation coefficient (cor) | ≥ 0.3 and *P* < 0.05 were considered to be significantly related to the immunocyte-infiltrating level. Immune infiltrating levels of tumor-infiltrating immune cells with different CNVs were compared. Moreover, survival analysis was conducted based on high- and low-infiltrating levels of immunocytes.

### Gene Set Enrichment Analysis (GSEA)

GSEA (27) was performed using TCGA-PRAD data to explore the potential functions of hub genes. Briefly, 495 PCs were divided into two groups (high- and low-expression groups) based on the median of hub gene expression levels. Further, “c2.cp.kegg.v7.0.symbols.gmt” was chosen as the reference gene sets. In this study, only KEGG signaling pathways reaching *P* < 0.05, |ES| > 0.6, gene size ≥100, and FDR < 25% were considered to be significantly enriched.

### Identification of Candidate Molecule Drugs

Connectivity map (CMap) (28) is an online webtool to identify molecule drugs showing high correlation to diseases (https://portals.broadinstitute.org/cmap/), which was applied to the overlapped immune- and stromal-related DEGs in this study to explore molecule drugs with strong correlations to PC. Molecule drugs with the number of instances *(n)* >5 and *P* < 0.05 were removed. Moreover, small molecule drugs with |mean| ≥ 0.40 were considered to possess significant therapeutic potential to treat PC.

## Results

### Immune and Stromal Scores Are Associated With Clinical Features and RFS of PC Patients

**Figure 2:**
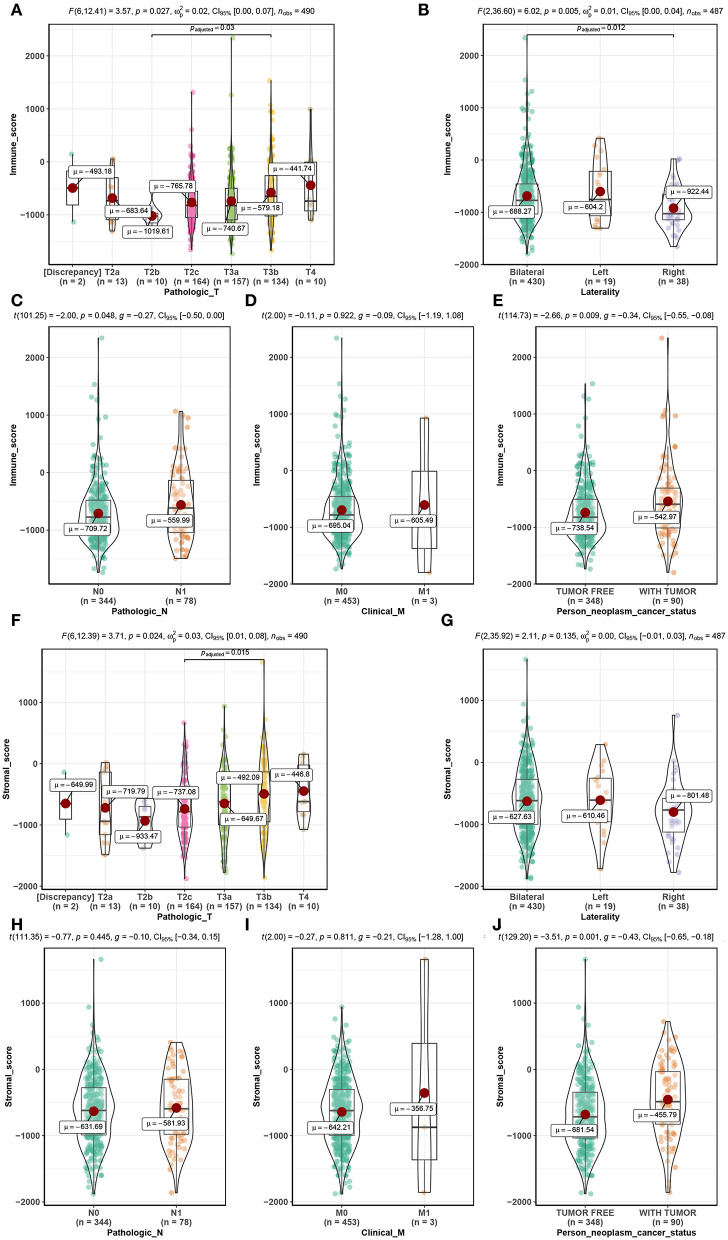
Distribution of immune scores of pathologic T **(A)**, laterality **(B)**, pathologic N **(C)**, clinical M **(D)**, and person neoplasm cancer status **(E)**. Distribution of stromal scores of pathologic T **(F)**, laterality **(G)**, pathologic N **(H)**, clinical M **(I)**, and person neoplasm cancer status **(J)**.

Among the 495 PCs downloaded from the TCGA database, 69.5% (*n* = 344) samples were pathological N0, and 15.8% (*n* = 78) samples were pathological N1. As for the pathological T, patients with T2a, T2b, T2c, T3a, T3b, and T4 accounted for 2.6% (*n* = 13), 2.0% (*n* = 10), 33.1% (*n* = 164), 31.7% (*n* = 157), 27.1% (*n* = 134), and 2.0% (*n* = 10), respectively. Clinical M0 accounted for 91.5% (*n* = 453), while clinical M1 accounted for 0.6% (*n* = 3). Bilateral tumors accounted for 86.9% (*n* = 430) of cases, while left and right tumors accounted for 3.8% (*n* = 19) and 7.7% (*n* = 38), respectively. Regarding neoplasm cancer status, tumor-free patients accounted for 70.3% (*n* = 348) of the total number, and 18.2% (*n* = 90) of cases had tumors. Additionally, immune scores ranged from −1795.98 to 2339.39 and stromal scores ranged from −1877.60 to 1659.96 (Table S1). Further analysis demonstrated that the immune score was significantly associated with pathological T (*F* = 3.570, *P* = 0.027, Figure 2A), laterality (*F* = 6.020, *P* = 0.005, Figure 2B), pathological N (*t* = −2.000, *P* = 0.048, Figure 2C) and neoplasm cancer status (*t* = −2.660, *P* = 0.009, Figure 2E). Moreover, stromal score was significantly related to pathological T (*F* = 3.710, *P* = 0.024, Figure 2F) and neoplasm cancer status (*t* = −3.510, *P* = 0.001, Figure 2J). Unfortunately, stromal score did not show significant relationship with laterality (Figure 2G), pathologic N (Figure 2H), and clinical M (Figure 2I). RFS survival analyses showed a trend of worse RFS in PC patients with high immune scores (*P* = 0.110, Figure S1B) or high stromal scores (*P* = 0.190, Figure S1D). No association between immune scores or stromal scores was found with OS in PC patients (Figures S1A,C).

### Immune- and Stromal-Related DEGs Identified in PC Cases

**Figure 3:**
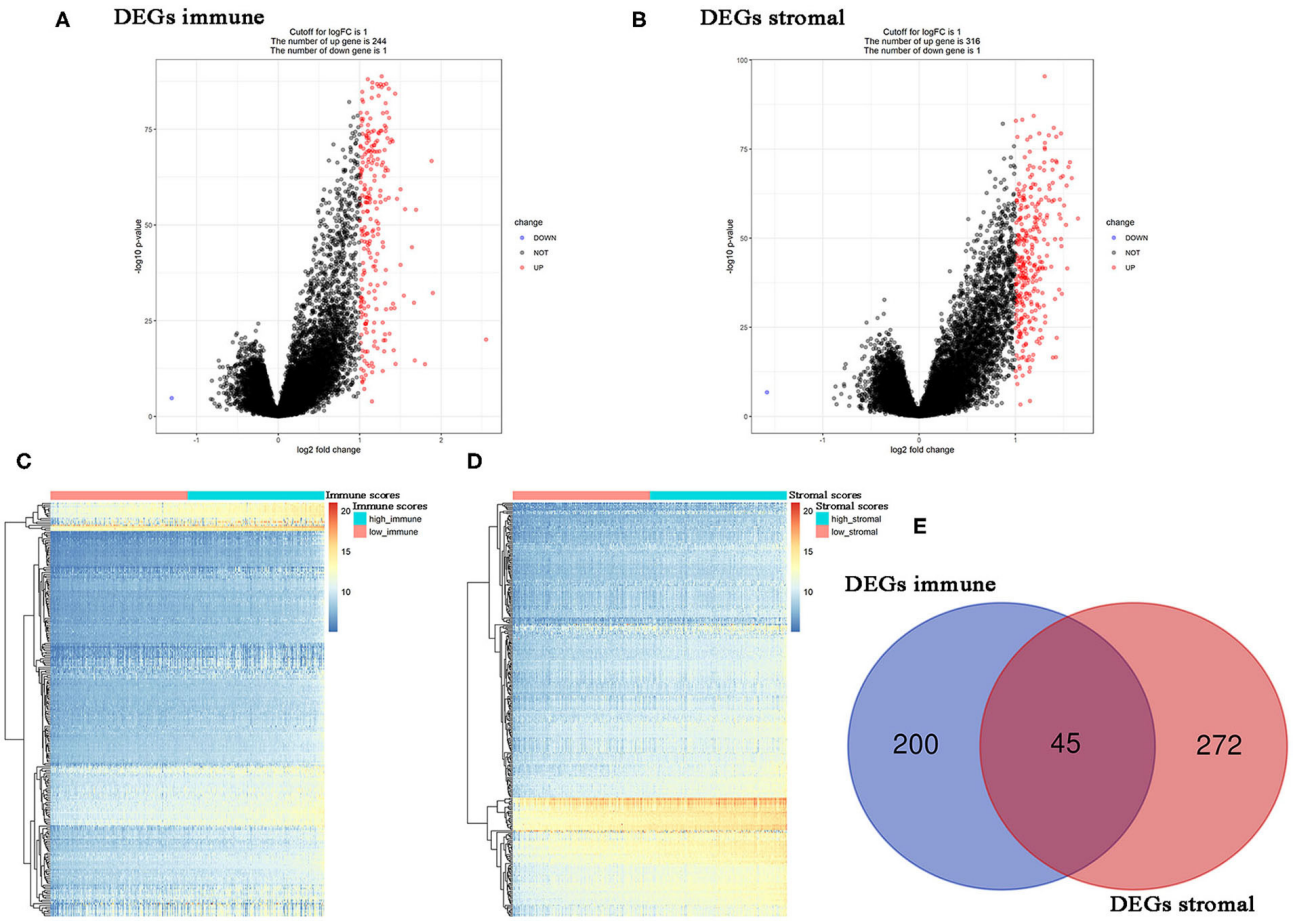
Differentially expressed genes (DEGs) analysis in PC. **(A)** Volcano plot visualizing the immune-related DEGs. **(B)** Volcano plot visualizing the stromal-related DEGs. **(C)** Heatmap of immune scores of high score vs. low score (*P* < 0.05, fold change >1). **(D)** Heatmap of stromal scores of high score vs. low score (*P* < 0.05, fold change >1). **(E)** Identification of common DEGs between immune-related DEGs and stromal-related DEGs.

**Figure 4:**
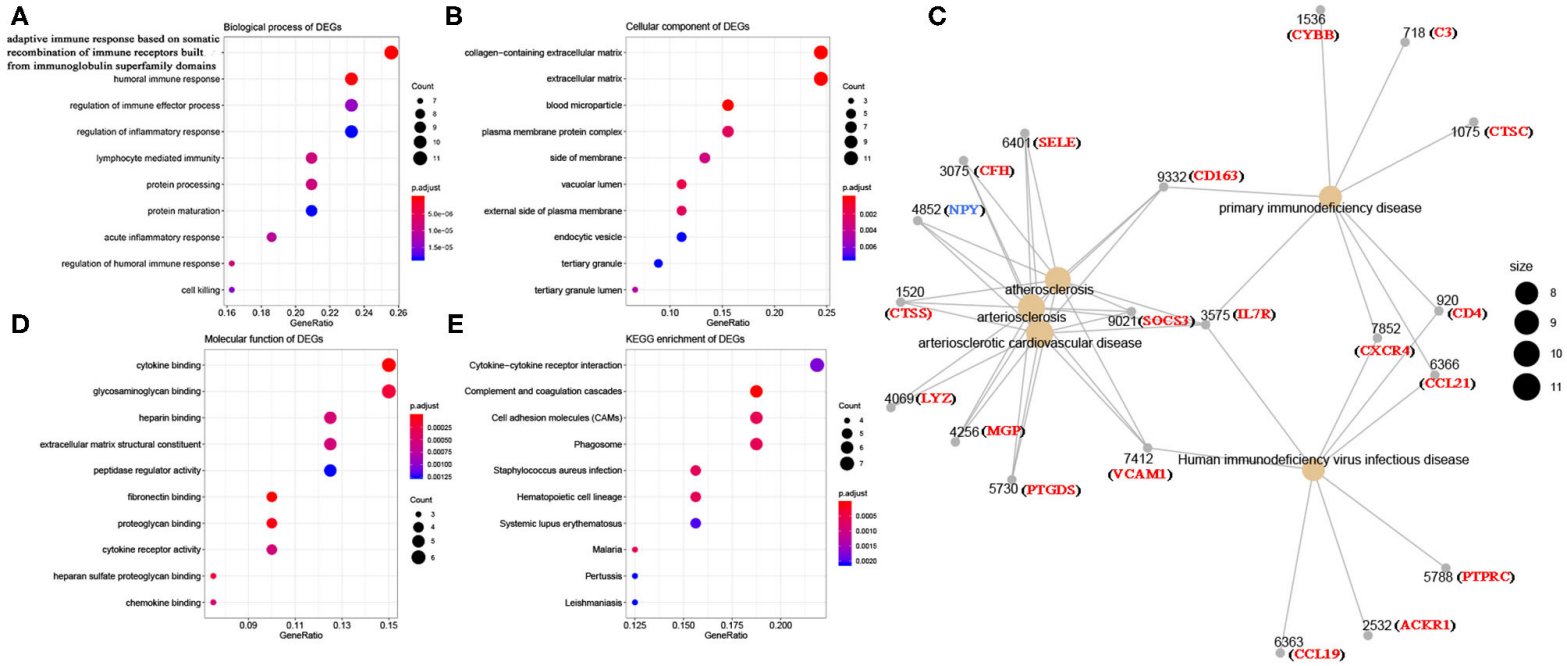
Bioinformatics analysis of 45 DEGs associated with immune score and stromal score. **(A)** Biological process of DEGs. **(B)** Cellular component of DEGs. **(C)** Disease ontology of DEGs. **(D)** Molecular function of DEGs. **(E)** KEGG enrichment of DEGs.

Using R package “limma,” 245 DEGs were found to be associated with immune score (244 up-regulated and 1 down-regulated) (Figures 3A,C, Table S2). Similarly, 371 stromal-related DEGs were selected, including 316 up-regulated DEGs and 1 down-regulated DEGs (Figures 3B,D, Table S3). Finally, 45 overlapping immune- and stromal-related DEGs were selected for subsequent analysis (Figure 3E). To outline the potential function of the DEGs, we performed functional enrichment analysis of the 45 DEGs. GO analysis demonstrated that the 45 DEGs were significantly enriched in 209 BPs (Table S4), 14 CCs (Table S5), and 21 MFs (Table S6) including humoral immune response, cell killing, extracellular matrices, and chemokine activities (Figure 4). Moreover, DEGs were significantly correlated with 14 KEGG signaling pathways (Table S7), including cytokine-cytokine receptor interaction, complement and coagulation cascades, and cell adhesion molecules (CAMs) (Figure 4E). Furthermore, DO analysis demonstrated that the 45 DEGs were highly associated with arteriosclerotic cardiovascular disease, arteriosclerosis, human immunodeficiency virus infectious disease, primary immunodeficiency disease, and atherosclerosis (Figure 4C, Table S8).

### Complement C7 as Prognostic Biomarker

**Figure 5:**
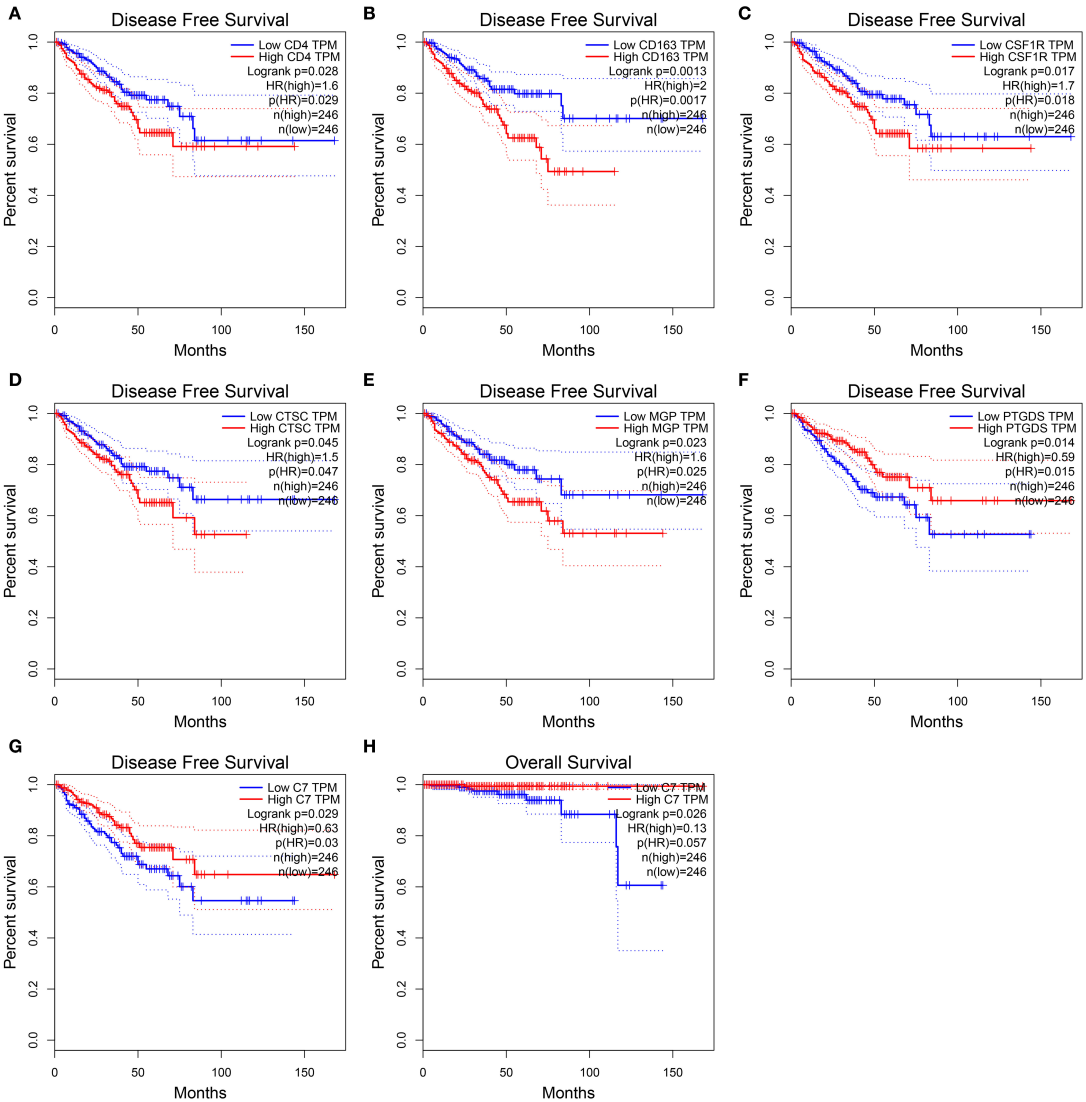
Disease-free survival analyses on CD4 **(A)**, CD163 **(B)**, CSF1R **(C)**, CTSC **(D)**, MGP **(E)**, PTGDS **(F)**, C7 **(G)** based on the TCGA-PRAD data. Overall survival analyses on C7 **(H)** based on the TCGA-PRAD data.

In this study, two different survival analyses, including OS and RFS, were performed for hub gene identification among 45 DEGs (Table S9). Seven genes, including CD4 (HR = 1.600, *P* = 0.028, Figure 5A), CD163 (HR = 2.000, *P* = 0.001, Figure 5B), CSF1R (HR = 1.700, *P* = 0.017, Figure 5C), CTSC (HR = 1.500, *P* = 0.045, Figure 5D), MGP (HR = 1.600, *P* = 0.023, Figure 5E), PTGDS (HR = 0.590, *P* = 0.014, Figure 5F), and C7 (HR = 0.630, *P* = 0.029, Figure 5G) were significantly correlated with RFS of PC patients. However, of the 45 DEGs, only C7 showed a significant *P*-value in OS analysis (HR = 0.130, *P* = 0.026, Figure 5H), indicating this gene as potential prognostic biomarker for further validation. Based on GEPIA, C7 expression in PCs was found to be significantly lower compared to normal tissues (Figure S2A). Furthermore, C7 expression was lower in higher clinical T stage (*F* = 4.74, *P* = 0.012, Figure S2C) and pathological T stage (*F* = 13.30, *P* = 0.007, Figure S2D) using GSE70770.

### Hub Gene Validation

**Figure 6:**
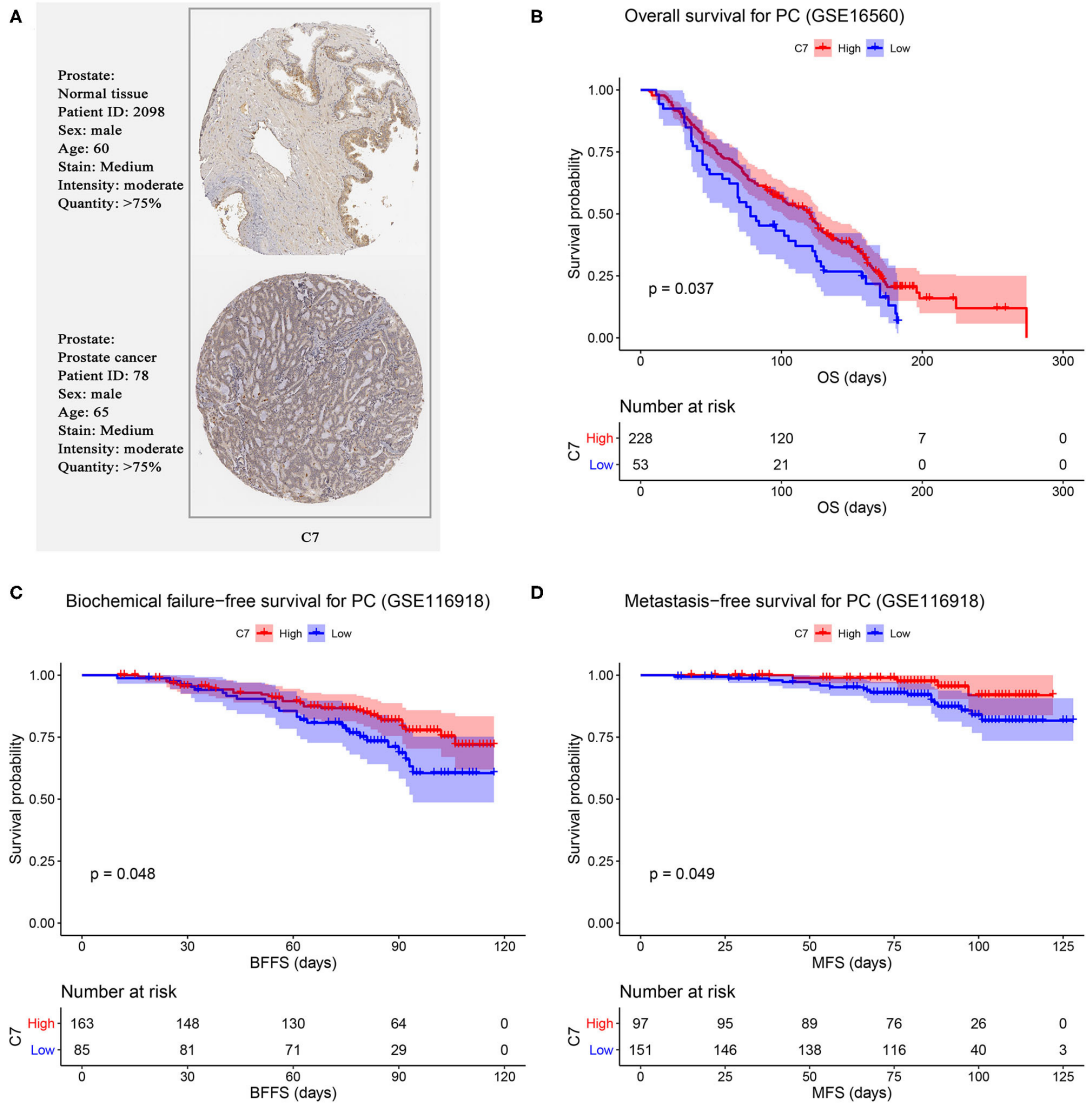
**(A)** Validation of hub genes in translational level by The Human Protein Atlas database (IHC). Survival analysis of the association between C7 expression and overall survival **(B)**, biochemical failure free survival **(C)**, and metastasis free survival **(D)** time in PC.

Using PC data from the Oncomine database (including 12 PC datasets), C7 mRNA expression tended to be lower in PCs than normal tissues (Figure S3). As shown in Figure 6A, no significant difference was found in C7 protein expression between PCs and normal tissues. In evaluating hub gene expression in various cancer cell lines by GEPIA and CCLE, C7 expression was found to differ in these tumor types. C7 expression was significantly lower in 15 cancer types compared with corresponding normal tissues (Figure S4A). Moreover, C7 mRNA expression in the prostate ranked as the bottom fifth across all the types of cancer cell lines (Figure S4B). Further, the CNV level of C7 in the prostate was the fifteenth highest of all cancer types (Figure S4C).

We further validated the prognostic value of C7 by performing survival analysis. PC patients with high expression of C7 had better OS (*P* = 0.037, Figure 6B). Similarly, low expression of C7 caused worse BFFS (*P* = 0.048, Figure 6C) and MFS (*P* = 0.049, Figure 6D), indicating that C7 was significantly associated with survival and prognosis of PC patients.

### C7 Genetic Alteration

**Figure 7:**
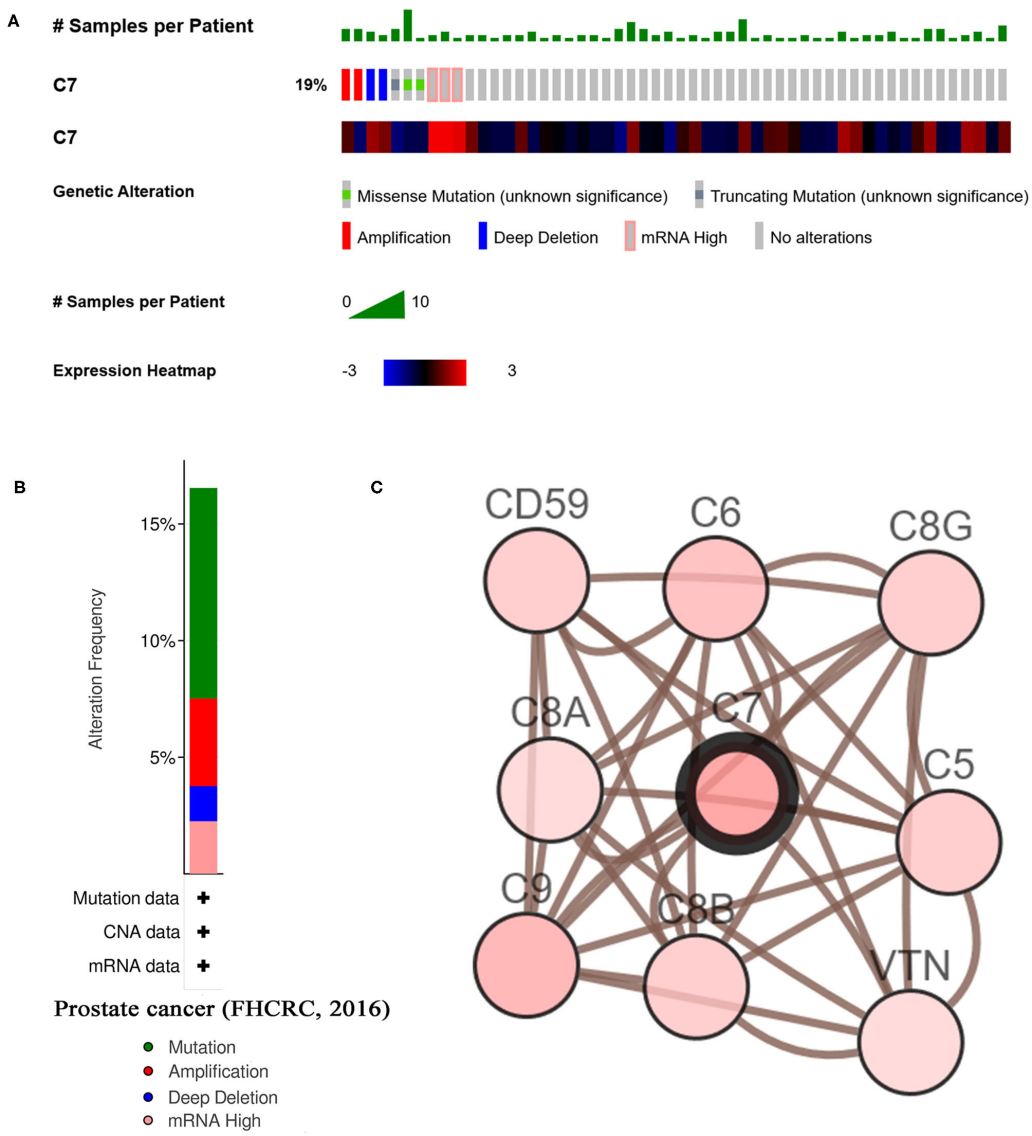
A summary of mutations and CNVs of hub genes. **(A)** Genetic alterations associated with C7 and expression heatmap of C7 based on the data from FHCRC. **(B)** The total alteration frequency of C7 in FHCRC is illustrated. **(C)** The network contains 9 nodes, including C7 and the 8 most frequently altered neighbor genes. Relationship between hub gene and tumor drugs is also illustrated.

As shown in Figure 7B, C7 was altered in 26 (19%) of 136 PC patients (Figure 7A). The main type of alteration was missense mutation (Figure 7B). Figure 7C showed the network containing nine nodes, including C7 and 8, consisted of the most altered neighbor genes. Currently, no drugs target C7, suggesting that C7 might be a novel therapeutic target for PC treatment.

### Relationships Between C7 and Clinical Features

**Figure 8:**
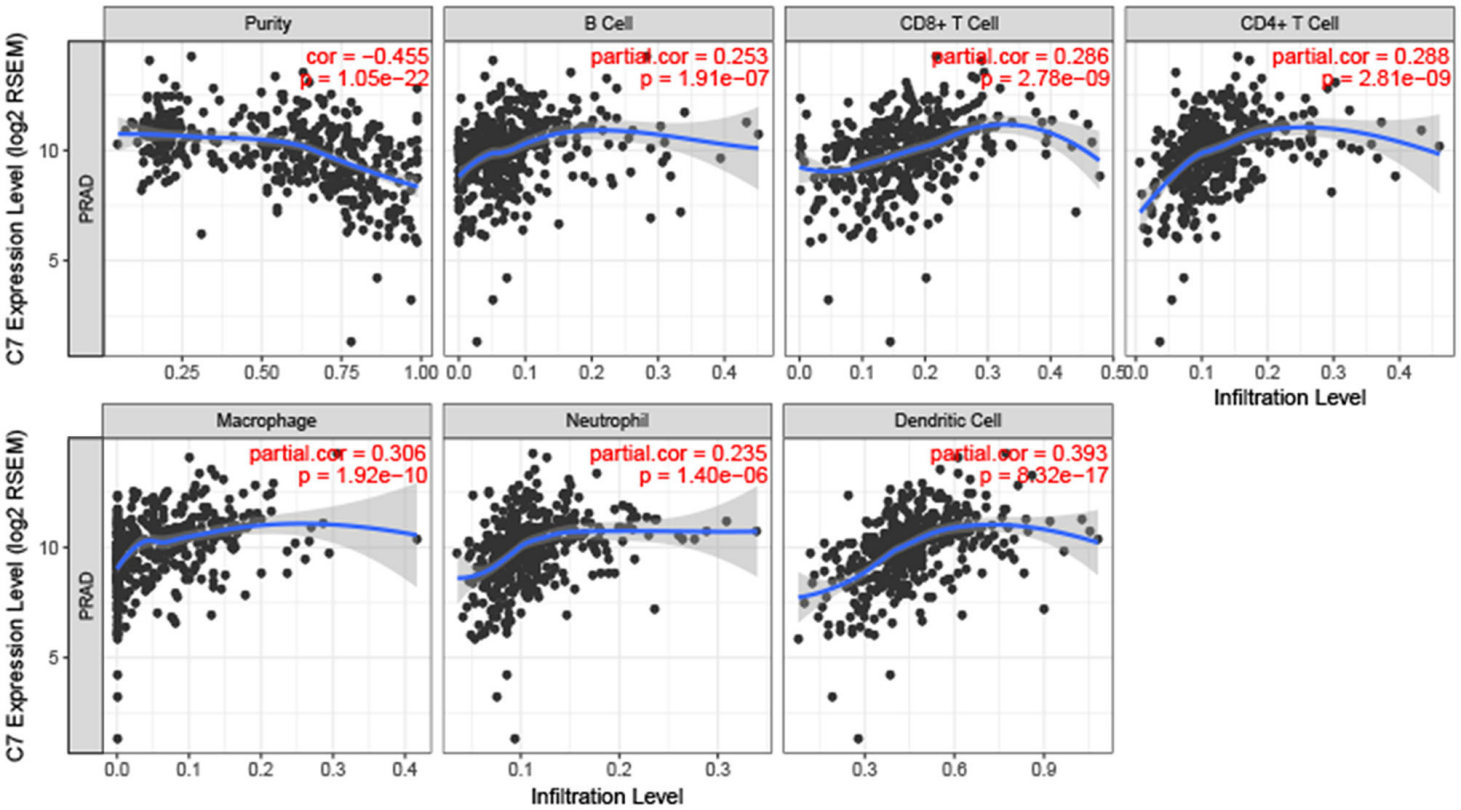
Correlation of C7 expression with immune infiltration level in PC. C7 expression was positively related to macrophages and dendritic cells meanwhile negatively associated with tumor purity.

To explore the correlation between C7 expression and clinical factors, Table 1 showed that C7 expression was significantly associated with stromal score (*P* < 0.001), immune score (*P* < 0.001), clinical T (*P* = 0.044), and pathological N (*P* = 0.004). As shown in Figure S5A, C7 (Hazard Ratio = 0.589, 95% CI of ratio: 0.390–0.890, *P* = 0.012), Gleason score (Hazard Ratio = 2.959, 95% CI of ratio: 1.337–6.549, *P* = 0.007), clinical T (Hazard Ratio = 3.253, 95% CI of ratio: 1.350–7.839, *P* = 0.009), and clinical N (Hazard Ratio = 58.924, 95% CI of ratio: 6.470–536.610, *P* < 0.001) were shown to be associated with OS by univariate Cox analysis. Following adjustments to other features, C7 (Hazard Ratio = 0.853, 95% CI of ratio: 0.504–1.443, *P* = 0.553) did not show a strong prognostic value. However, the Gleason score was shown to be significant (Hazard Ratio = 3.010, 95% CI of ratio: 1.029–8.810, *P* = 0.044) by multivariate Cox analysis (Figure S5B). Due to the association of immune infiltration level with survival and prognosis in cancers, the correlation between C7 and immune infiltration level was assessed. As shown in Figure 8, C7 expression was positively associated with macrophages (cor = 0.306, *P* = 1.92E-10) and dendritic cells neutrophils (cor = 0.393, *P* = 8.32E-17). Moreover, C7 expression was negatively correlated to tumor purity (cor = −0.455, *P* = 1.05E-22), demonstrating in part a significant correlation between C7 and immune infiltration in PC.

**Table 1:** Associations between C7 expression and clinicopathological factors of patients with PC (based on TCGA-PRAD).

**Characteristics**	**C7 expression**		**Chi-square**	***P***
	**Low**	**High**		
	***n* = 248**	***n* = 247**		
**Stromal score**				
Low	185	63	119.29	<0.001
High	63	184		
**Immune score**				
Low	159	89	39.032	<0.001
High	89	158		
**Age**				
≤ 65	169	163	0.26	0.61
>65	79	84		
**Laterality**				
Bilateral	211	219	4.41	0.11
Left	8	11		
Right	25	13		
NA	4	4		
**Clinical M**				
M0	226	227	0	1
M1	2	1		
NA	20	19		
**Clinical T**				
T1	79	98	8.098	0.044
T2	93	79		
T3	32	21		
T4	2	0		
NA	42	49		
**Pathologic N**				
N0	167	177	8.363	0.004
N1	52	26		
NA	29	44		
**Pathologic T**				
T2	87	100	2.516	0.284
T3	155	136		
T4	4	6		
NA	2	5		
**Cancer status**				
Tumor free	167	181	0.513	0.474
With tumor	47	43		
NA	34	23		

### C7 Enrichment in Nine KEGG Signaling Pathways

To explore potential functions of C7, GSEA was conducted, showing that C7 was significantly correlated to nine KEGG signaling pathways, including the following: cytokine cytokine receptor interaction (nominal *P* = 0.006, |ES| = 0.682, gene size = 256, FDR = 8.634%), focal adhesion (nominal *P* = 0.002, |ES| = 0.666, gene size = 194, FDR = 8.233%), chemokine signaling pathway (nominal *P* = 0.017, |ES| = 0.636, gene size = 185, FDR = 8.814%), calcium signaling pathway (nominal *P* < 0.001, |ES| = 0.666, gene size = 177, FDR = 7.562%), JAK stat signaling pathway (nominal *P* = 0.006, |ES| = 0.615, gene size = 153, FDR = 7.443%), cell adhesion molecules cams (nominal *P* = 0.004, |ES| = 0.721, gene size = 128, FDR = 8.147%), axon guidance (nominal *P* < 0.001, |ES| = 0.629, gene size = 127, FDR = 8.492%), leukocyte transendothelial migration (nominal *P* = 0.004, |ES| = 0.624, gene size = 113, FDR = 7.997%), and vascular smooth muscle contraction (nominal *P* = 0.002, |ES| = 0.668, gene size = 112, FDR = 19.888%) (Table 2).

**Table 2:** Genet set enrichment analysis (GSEA) of C7.

**Name**	**SIZE**	**ES**	**NES**	**NOM p-val**	**FDR**
KEGG_CYTOKINE_CYTOKINE_RECEPTOR_INTERACTION	256	−0.68186	−1.5713	0.006198	0.086336
KEGG_FOCAL_ADHESION	194	−0.66639	−1.73353	0.002058	0.082326
KEGG_CHEMOKINE_SIGNALING_PATHWAY	185	−0.63571	−1.56574	0.016563	0.08814
KEGG_CALCIUM_SIGNALING_PATHWAY	177	−0.66613	−1.73198	0	0.075619
KEGG_JAK_STAT_SIGNALING_PATHWAY	153	−0.61527	−1.70465	0.005964	0.074439
KEGG_CELL_ADHESION_MOLECULES_CAMS	128	−0.72085	−1.59475	0.004107	0.081467
KEGG_AXON_GUIDANCE	127	−0.62915	−1.67698	0	0.084921
KEGG_LEUKOCYTE_TRANSENDOTHELIAL_MIGRATION	113	−0.6244	−1.63166	0.004167	0.079972
KEGG_VASCULAR_SMOOTH_MUSCLE_CONTRACTION	112	−0.66842	−1.79457	0.002024	0.198877

### Strong Therapeutic Potential Shown by Six Small Molecule Drugs

Highly associated molecule drugs were identified by CMap. In total, 12 molecule drugs were screened (Table 3), six of which, including amiloride (mean = 0.656, *n* = 5, *P* < 0.001), finasteride (mean = −0.513, *n* = 6, *P* = 0.006), metronidazole (mean = −0.430, *n* = 5, *P* = 0.006), kawain (mean = 0.502, *n* = 5, *P* = 0.009), guanabenz (mean = −0.419, *n* = 5, *P* = 0.017), and fluocinonide (mean = 0.469, *n* = 5, *P* = 0.043), were identified as potential novel drug candidates for PC treatment.

**Table 3:** Results of CMap analysis based on DEGs in PC.

**Cmap name**	**Mean**	***n***	**Enrichment**	***p***	**Specificity**	**%non-null**
Amiloride	0.656	5	0.814	0.00054	0	100
Dipyridamole	0.388	6	0.71	0.00169	0.0067	66
Finasteride	−0.513	6	−0.639	0.00636	0.1342	66
Metronidazole	−0.43	5	−0.69	0.00643	0.0345	60
Kawain	0.502	5	0.675	0.00949	0	80
Karakoline	−0.382	6	−0.6	0.01426	0	50
Guanabenz	−0.419	5	−0.632	0.01746	0.1308	60
0173570-0000	0.286	6	0.556	0.02886	0.1543	50
Dicycloverine	−0.365	5	−0.6	0.02924	0.0534	60
Hesperetin	0.352	5	0.582	0.03973	0.0325	60
Fluocinonide	0.469	5	0.578	0.04232	0.0821	60
CP-690334-01	−0.383	8	−0.464	0.0434	0.1769	50

## Discussion

Tumor microenvironment (TME), the internal environment of the tumor for growth and proliferation, consists of non-cancerous cells (29), including fibroblasts, immune cells, cells that comprise the blood vessels, and secreted proteins produced by all cells present in the tumor (30). Two major cell types (immune and stromal cells) in the TME have been reported to have strong correlations with tumor prognosis (30). With the development of precision medicine, immunotherapy has been one of the most effective treatment methods for malignancies, which mainly stimulates autoimmune or alloimmune cells in patients to improve symptoms, prolong survival, and improve prognosis (31, 32).

Prostate cancer (PC) is one of the most common malignancies in the urinary system and the second most common tumor in men. Currently, surgical resection plus radiotherapy and hormone therapy are the standard treatment methods. Although this method is effective for early and localized PC treatment, PC develops quickly, leading to metastasis. Also, nowadays there are some treatment available for advanced PC, and nearly all have resulted in prolongation of survival. Unfortunately, a recent study has shown that the 5-year survival rate of PC was <30% (33). Because of the poor prognosis of PC, at the same time taking into account the effectiveness and safety of immunotherapy, exploring novel prognostic biomarkers and therapeutic targets for immunotherapy is needed.

In this study, we applied the ESTIMATE algorithm to calculate immune and stromal scores of PC cases from TCGA-PRAD data. Following, 45 overlapping immune- and stromal-related DEGs were chosen for further analysis. Among them, only one gene C7 was significantly associated with OS and RFS of PC patients, which was considered as a hub gene in this study. Follow-up analysis confirmed that C7 expression was lower in PCs compared to normal tissues. Moreover, Oncomine analysis demonstrated that C7 mRNA expression was higher in normal tissues compared to that in PCs, which was consistent with the TCGA-PRAD data. Furthermore, based on GSE29609, results suggested that C7 expression was significantly associated with OS and BFFS (and MFS), demonstrating that C7 was a powerful prognostic biomarker in PC.

Because immune infiltration level showed a strong correlation with survival in tumors (34), we explored the association between C7 expression level and immune infiltration level in PC by TIMER. C7 expression was found to be significantly negatively related to tumor purity while positively associated with macrophages and dendritic cells neutrophils. Thus, C7 might be a potential therapeutic target for immunotherapy in PC.

C7 can form a membrane attack complex together with complement components C5b, C6, C8, and C9 (35). This complex worked as part of the terminal complement pathway of the innate immune system (35). Moreover, C7 expressed on the cell membrane acted as a regulator of the excessive proinflammatory reaction as reported (36). In addition, Li et al. demonstrated that low expression of C7 in non-small cell lung cancer (NSCLC) might act as a tumor inhibitor, which was associated with tumor progression and prognosis (36). In detail, they firstly found the C7 mRNA content descended gradually in normal, benign, borderline and malignant ovarian tissues based on the fluorescent *in situ* hybridization assay (Consistent downward tendency of C7 mRNA expression was also found in the course of lung tumor progression). Then they attempted to explore the prognostic potential of C7 by using survival analysis. Unfortunately, they only found that NSCLC patients with decreased expression of C7 had a worse outcome. Finally, some experiments were done to demonstrate that C7 overexpression inhibited proliferation of NSCLC cells *in vitro*. Although our study was not based on experiments like theirs, similarly, in our study, we found low expression of C7 in PC compared to normal tissues by using some authoritative public databases including GEO, Oncomine, TCGA. Furthermore, we demonstrated that PC patients with decreased expression of C7 had a worse outcome by the public databases we could use. Therefore, we hypothesized that C7 might be a potential tumor suppressor associated with survival and prognosis of PC and might be a new therapeutic target for PC immunotherapy.

Despite the study design and strict filtering criteria at each step, there were some limitations in this study. First, although we validated the hub gene systematically and comprehensively, C7 did not show significant differences at the protein level. Thus, protein expression will be assessed by western blotting in future studies. Second, although we tried our best to validate our findings by using the public databases we could use, this study was lack of experiment. Thus, we will conduct C7 overexpression and colony formation assay in PC and investigate the cell derivation of C7 (local or by infiltrating neutrophils) in the shore future. Third, no clinical trials have been conducted to assess the prognostic value of C7 in this study, which is the focus of upcoming studies.

## Conclusion

In summary, we identified 45 TME-related DEGs (associated with immune and stromal scores) by applying the ESTIMATE algorithm. A potential immune-related prognostic biomarker named C7 was further screened and validated, which might function as a tumor suppressor in prostate cancer. Moreover, six small molecule drugs were identified that showed strong therapeutic potential for PC treatment.

## Data Availability Statement

The data that support the findings of this study are openly available in Gene Expression Omnibus (GEO) database at http://www.ncbi.nlm.nih.gov/geo/ and The Cancer Genome Atlas (TCGA) database at https://genome-cancer.ucsc.edu/.

## Author Contributions

T-ZL, ZC, and XY conceived and designed the study. ZC, XY, and G-WD performed the analysis procedures. G-WD, XY, ZC, R-JZ, KT, X-JB, H-HW, G-FX, and T-ZL analyzed the results. T-ZL and XY contributed analysis tools. ZC contributed to the writing of the manuscript. All authors reviewed the manuscript.

## Conflict of Interest

The authors declare that the research was conducted in the absence of any commercial or financial relationships that could be construed as a potential conflict of interest.

## Supplementary Material

Figure S1 Survival analysis of the association between immune score and overall survival **(A)**, disease-free survival **(B)** time in PC. Survival analysis of the association between stromal score and overall survival **(C)**, disease-free survival **(D)** time in PC.

Figure S2 **(A)** Expression comparison of candidate hub genes in PCs and normal tissues. **(B)** Stage plots of C7 across different T stages in GSE116918. **(C)** Stage plots of C7 across different clinical T stages in GSE70770. **(D)** Stage plots of C7 across different pathology T stages in GSE70770.

Figure S3 Oncomine database analyses. comparison of C7 mRNA expression across 12 analyses of PC.

Figure S4 **(A)** Comparison of C7 mRNA expression between tumors and normal tissues across all the types of cancers from TCGA data. Cancer Cell Line Encyclopedia analysis of C7 mRNA expression **(B)** and copy number variation level **(C)** in prostate (black boxes) and other cancer cell lines.

Figure S5 Forest plot summary of analyses of OS. Univariable **(A)** and multivariable analyses **(B)** of the stromal score, immune score, C7, age, laterality, gleason score, clinical T, clinical M, pathologic T, and pathologic N on all 495 PC patients.

Table S1 Stromal score and immune score of each patient with PRAD (based on TCGA-PRAD).

Table S2 Differentially expressed genes (DEGs) between low-immune samples and high-immune samples.

Table S3 Differentially expressed genes (DEGs) between low-stromal samples and high-stromal samples.

Table S4 GO biological processes of common DEGs.

Table S5 GO cellular component of common DEGs.

Table S6 GO molecular function of common DEGs.

Table S7 KEGG enrichment of common DEGs.

Table S8 Disease Ontology of common DEGs.

Table S9 Survival analysis of common DEGs.
