# Supplementary figures and images for: Complement C7 (C7), a Potential Tumor Suppressor, Is an Immune-Related Prognostic Biomarker in Prostate Cancer (PC)
Source: Front Oncol. 2020 Aug 25;10:1532. doi: 10.3389/fonc.2020.01532 (PMC7477933; doi:10.3389/fonc.2020.01532)

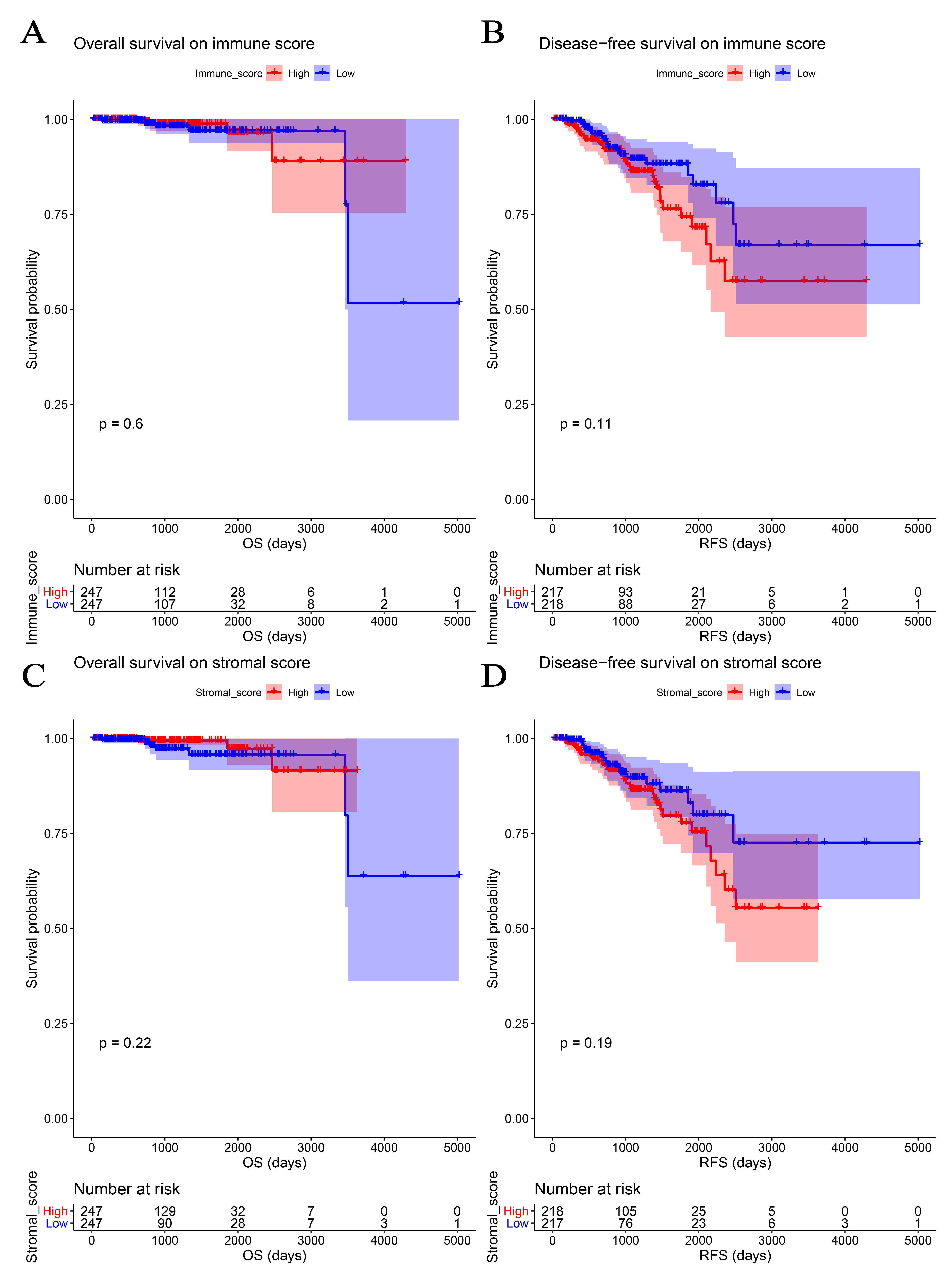

Supplement: Figure S1 — Survival analysis of the association between immune score and overall survival (A), disease-free survival (B) time in PC. Survival analysis of the association between stromal score and overall survival (C), disease-free survival (D) time in PC. [file Image_1.TIF]

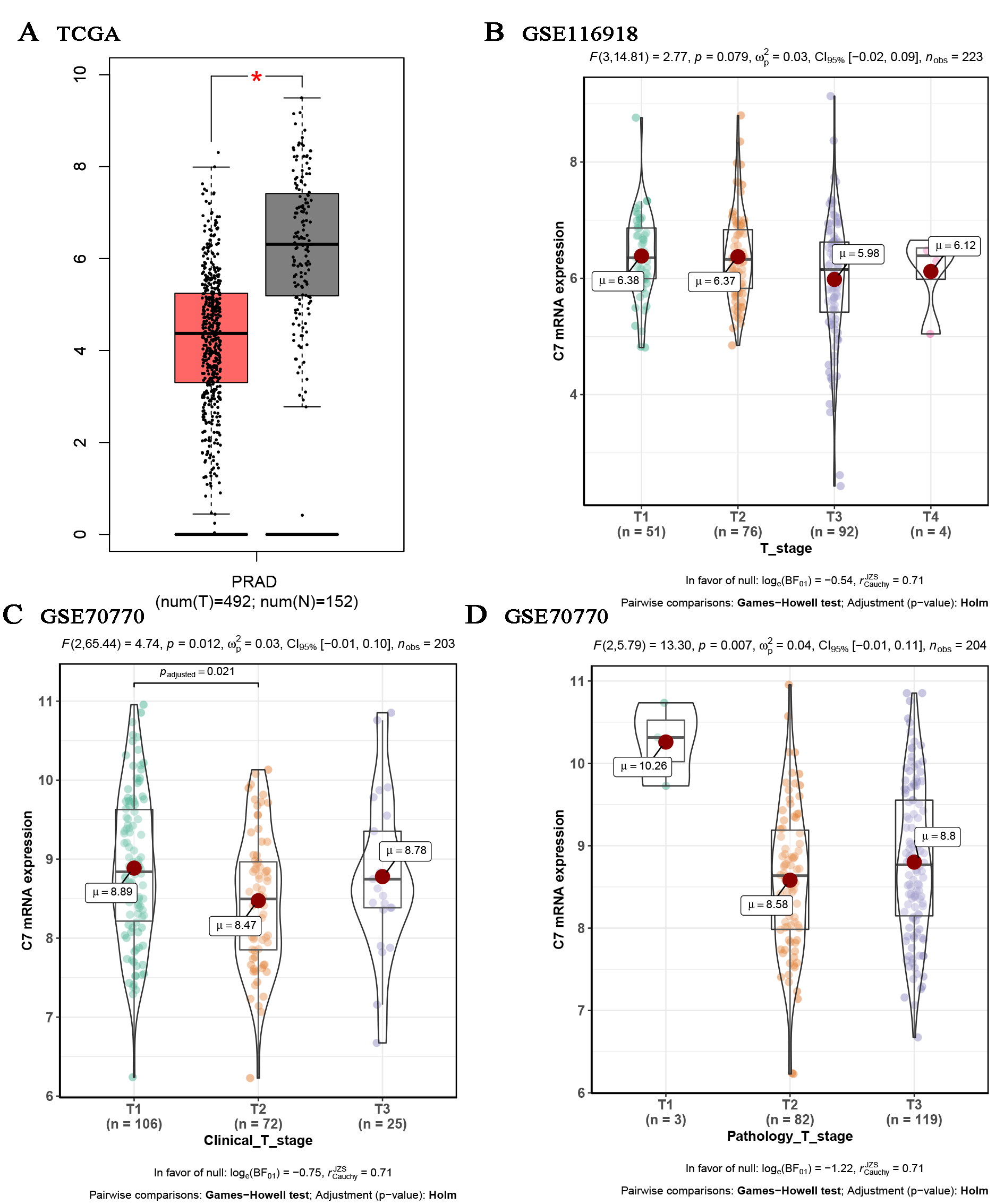

Supplement: Figure S2 — (A) Expression comparison of candidate hub genes in PCs and normal tissues. (B) Stage plots of C7 across different T stages in GSE116918. (C) Stage plots of C7 across different clinical T stages in GSE70770. (D) Stage plots of C7 across different pathology T stages in GSE70770. [file Image_2.TIF]

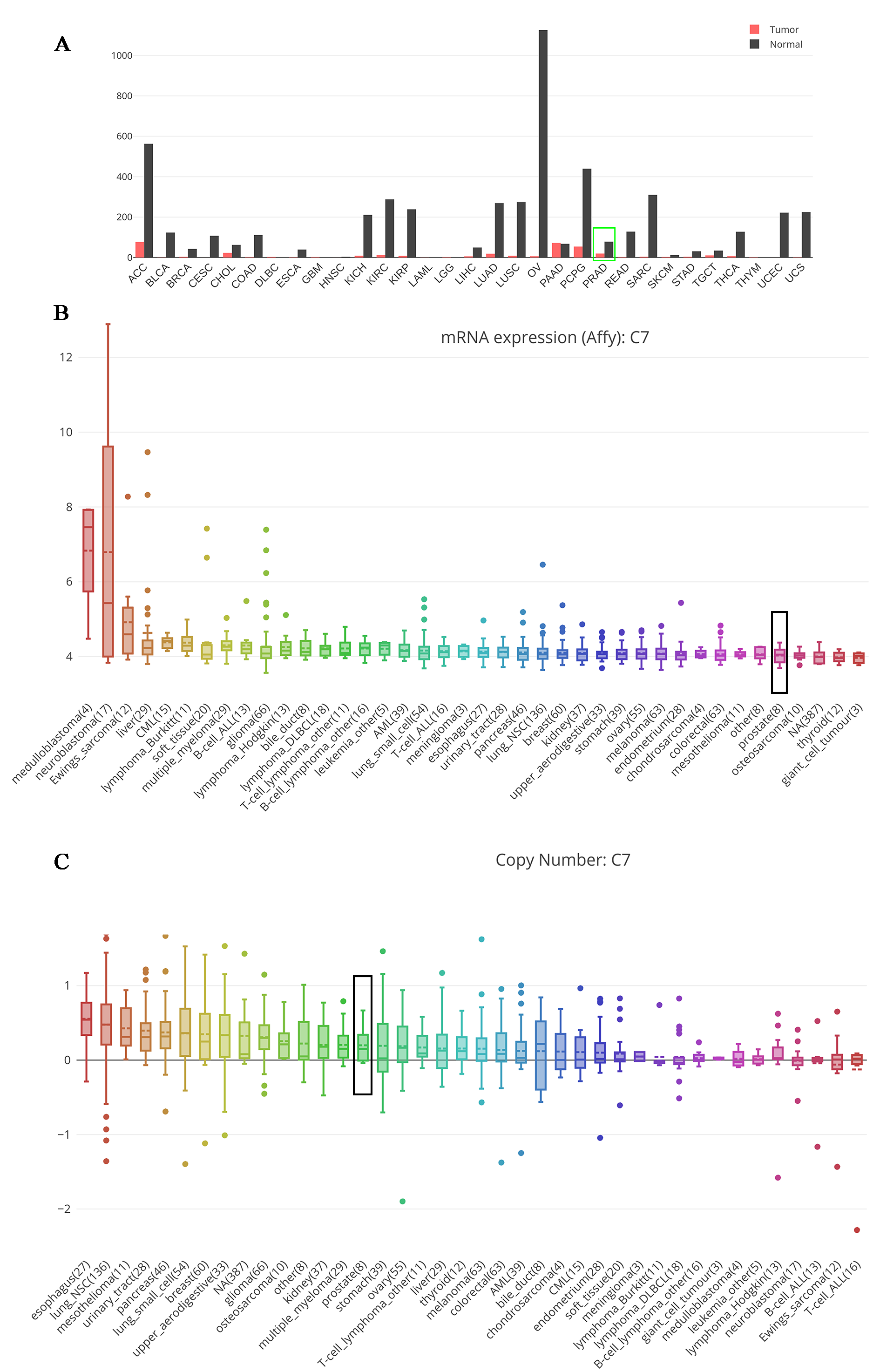

Supplement: Figure S4 — (A) Comparison of C7 mRNA expression between tumors and normal tissues across all the types of cancers from TCGA data. Cancer Cell Line Encyclopedia analysis of C7 mRNA expression (B) and copy number variation level (C) in prostate (black boxes) and other cancer cell lines. [file Image_4.TIF]

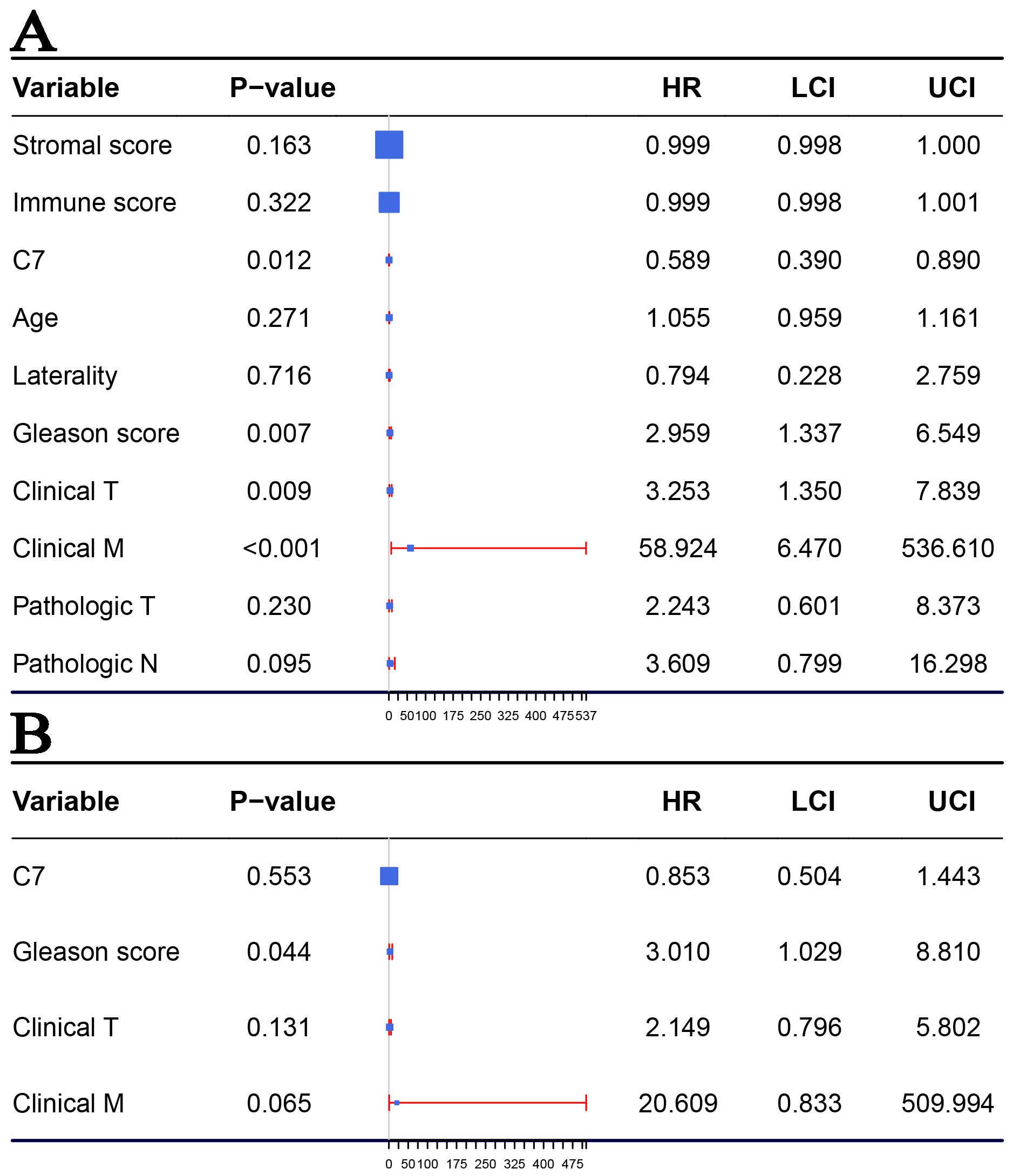

Supplement: Figure S5 — Forest plot summary of analyses of OS. Univariable (A) and multivariable analyses (B) of the stromal score, immune score, C7, age, laterality, gleason score, clinical T, clinical M, pathologic T, and pathologic N on all 495 PC patients. [file Image_5.TIF]
